# Bradykinin-induced angioedema in the emergency department

**DOI:** 10.1186/s12245-022-00408-6

**Published:** 2022-03-26

**Authors:** Jacques Hébert, Jean-Nicolas Boursiquot, Hugo Chapdelaine, Benoit Laramée, Marylin Desjardins, Rémi Gagnon, Nancy Payette, Oleksandra Lepeshkina, Matthieu Vincent

**Affiliations:** 1 CHU de Québec, Université Laval, Québec, Canada; 2 CHU de Montréal, Université de Montréal, Montréal, Canada; 3 Polyclinique Médicale Pierre-Le Gardeur, Terrebonne, Canada; 4 McGill University, Montréal, Canada; 5 CHU Sainte-Justine, Université de Montréal, Montréal, Canada; 6 Université de Sherbrooke, Sherbrooke, Canada; 7 Hôpital Charles-Le Moyne, Greenfield Park, Canada

**Keywords:** Bradykinin, Histamine, Angioedema, Emergency

## Abstract

**Background:**

Acute airway angioedema commonly occurs through two distinct mechanisms: histamine- and bradykinin-dependent. Although they respond to distinct treatments, these two potentially life-threatening states present similarly. Poor recognition of the bradykinin-dependent pathway leads to treatment errors in the emergency department (ED), despite the availability of multiple pharmacologic options for hereditary angioedema (HAE) and other forms of bradykinin-induced angioedema. Here, we consider the pathophysiology and clinical features of bradykinin-induced angioedema, and we present a systematic literature review exploring the effectiveness of the available therapies for managing such cases.

**Methods:**

PubMed searches using ‘emergency’, ‘bradykinin’ and various therapeutic product names identified studies reporting the efficacy of treatments for bradykinin-induced angioedema in the ED setting. In all, 22 studies met prespecified criteria and are analysed here.

**Findings:**

Whereas histamine-induced angioedema has a faster onset and often presents with urticaria, bradykinin-induced angioedema is slower in onset, with greater incidence of abdominal symptoms. Acute airway angioedema in the ED should initially be treated with anaphylactic protocols, focusing on airway management and treatment with epinephrine, antihistamine and systemic steroids. Bradykinin-induced angioedema should be considered if this standard treatment is not effective, despite proper dosing and regard of beta-adrenergic blockade. Therapeutics currently approved for HAE appear as promising options for this and other forms of bradykinin-induced angioedema encountered in the ED.

**Conclusion:**

Diagnostic algorithms of bradykinin-induced angioedema should be followed in the ED, with early use of approved therapies to improve patient outcomes.

## Background

Obstruction of the upper airway due to angioedema is a life-threatening event. Most such attacks are caused by an allergic reaction mediated by histamine. However, a non-allergic form mediated by bradykinin is also seen and may be mistaken for histamine-induced angioedema.

Because these types of angioedema respond to distinct treatments, prompt diagnosis is essential for reversing a potentially fatal airway attack. This article presents a clinical algorithm adapted from evidence-based guidelines, addressing the management of bradykinin-induced angioedema in the ED, as well as a systematic literature analysis examining the treatment of bradykinin-induced angioedema in the ED setting.

## Methods

A systematic literature search in PubMed was conducted to identify articles addressing bradykinin-induced angioedema treatment in the ED setting, using the search terms ‘plasma-derived C1-INH’ or ‘recombinant C1-INH’ or ‘ecallantide’ or ‘icatibant’ or ‘fresh frozen plasma’ and ‘emergency’ and ‘bradykinin’. Of 137 prospective and retrospective studies, case series and case reports identified, 122 were excluded because they did not describe ED treatment of bradykinin-induced events or did not report treatment efficacy. Seven additional studies were identified by targeted searches or scanning bibliographies, for a total of 22 studies analysed below.

### What is the pathophysiology of bradykinin-induced angioedema and how does it differ from histamine-induced angioedema?

**Fig. 1:**
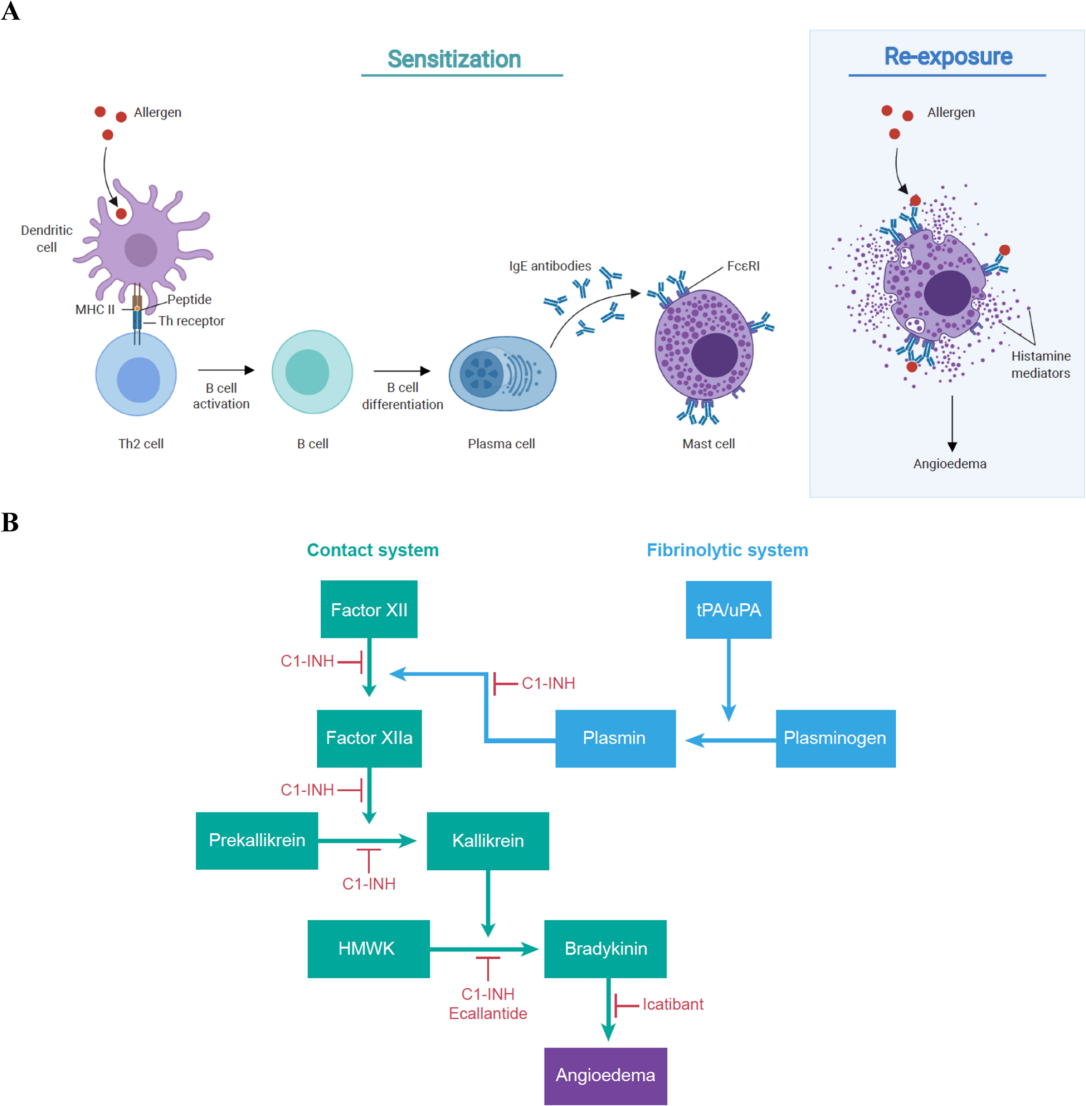
Mechanisms of angioedema [2, 6–8]. **A** Histamine induced. Upon exposure to an allergen, it is taken up by antigen-presenting cells (e.g. dendritic cells) and proteolyzed to produce small peptides. These peptides are then presented with major histocompatibility (MHC) class II antigen as a complex on the cell surface and recognised by T-helper (Th) lymphocyte receptors. This leads to the activation of T cells and release of Th2 cytokines that promote the differentiation of B cells to plasma cells and the production of specific IgE antibodies that recognise the original antigen. These antibodies bind to high-affinity IgE receptor FcεRI on mast cells and persist for weeks, months or years. Upon re-exposure to the allergen, the allergenic peptide is recognised by these bound IgEs, activating the mast cells to release bioactive mediators such as histamine. Binding of histamine on selective receptors of the vascular endothelium causes vasodilation and increased permeability. Mast cells can also be activated and triggered to produce histamine mediators through non-IgE-mediated response. **B** Bradykinin-induced. The contact pathway is initiated when factor XII (or Hageman factor) binds to damaged tissue and converts to factor XIIa, which then converts prekallikrein to plasma kallikrein. Finally, kallikrein cleaves HMWK to form bradykinin, which binds B2 receptors on the vascular endothelium, triggering vasodilation and increased permeability. Plasmin from the fibrinolytic system can convert factor XII into factor XIIa, accelerating the bradykinin production from HMWK. Multiple biological and pharmacological inhibitors can be used to treat bradykinin-induced angioedema. C1-INH can act on multiple stages of the contact and fibrinolytic system to inhibit the production of bradykinin. Ecallantide is a kallikrein inhibitor that blocks the cleavage of HMWK into bradykinin, and icatibant is an antagonist that prevents bradykinin from binding to its receptor

Angioedema is characterised by the localised increase of vascular permeability and dilation, triggered by the release of mediators such as histamine or bradykinin [1–5]. The mechanism underlying histamine-induced (allergic) angioedema is shown in Fig. 1A [2, 6, 7]. During first exposure to an allergen, specific immunoglobulin E (IgE) antibodies sensitise mast cells by binding high-affinity IgE receptors (FcεRI). During a re-encounter, allergen binding to IgE-FcεRI complexes promotes release of histamine, which binds receptors on the vascular endothelium, triggering angioedema.

Bradykinin-induced angioedema may be hereditary, acquired or drug induced [1, 2, 4, 5]. Bradykinin is generated through the cleavage of high-molecular-weight kininogen (HMWK) by activated kallikrein as shown in Fig. 1B [2, 8]. Kallikrein activation occurs through the contact system via factor XII, which can be activated by plasmin through the fibrinolysis pathway. Bradykinin binds the bradykinin B2 receptor on the vascular endothelium, stimulating substance P release and inducing angioedema.

C1 esterase inhibitor (C1-INH), which acts as a brake on the complement system, blocks bradykinin overproduction via the contact system. Hereditary angioedema (HAE) arises from mutations in the gene encoding C1-INH, reducing its expression (type I) or function (type II) [1, 2, 4, 5, 9]. Nonhereditary angioedema is caused by C1-INH overconsumption, which can occur with lymphoproliferative or autoimmune disease. In drug-induced angioedema, bradykinin or substance P turnover is blocked due to inhibition of catabolic enzymes including angiotensin-converting enzyme (ACE), neprilysin, dipeptidyl peptidase 4, carboxypeptidase and aminopeptidase P [10].

### What are key clinical features that differentiate bradykinin-induced angioedema from histamine-induced angioedema?

Although there are no validated tests available to quickly distinguish between histamine- or bradykinin-induced angioedema in the ED setting, differences in the typical clinical presentation of these distinct conditions (Table 1) can help guide diagnosis [1, 2, 4, 5].

**Table 1:** Clinical presentation of histamine- and bradykinin-induced angioedema [1–4]

Types of angioedema	Histamine induced	Bradykinin induced
**Causes**	-Allergy (anaphylaxis) -Non-allergic -Spontaneous (idiopathic)	-Hereditary (HAE) -Acquired -Drug induced (e.g. ACEi)
**Presence of urticaria**	Possible	No
**Onset**	Rapid (min)	Slower (hours)
**Duration**	Hours	Days
**Abdominal pain**	Could happen	Frequent (by history)
**Asthmatic response**	Frequent	No
**Hypotension**	Common	No
**Response to standard treatment (epinephrine, antihistamines, corticosteroids)**	Effective	No or minimal response

Histamine-induced angioedema often presents with urticaria and other manifestations of anaphylaxis such as bronchospasm, wheezing and hypotension. Onset is rapid and attack duration may be brief. In contrast, bradykinin-induced angioedema is usually not associated with urticaria and tends to have slower onset, longer duration and involve abdominal symptoms. A defining feature that distinguishes between these two types of angioedema is that bradykinin-induced angioedema responds poorly, if at all, to epinephrine, antihistamines or corticosteroids [1–5].

### What is the current approach to identify and manage bradykinin-induced angioedema in the ED?

**Fig. 2:**
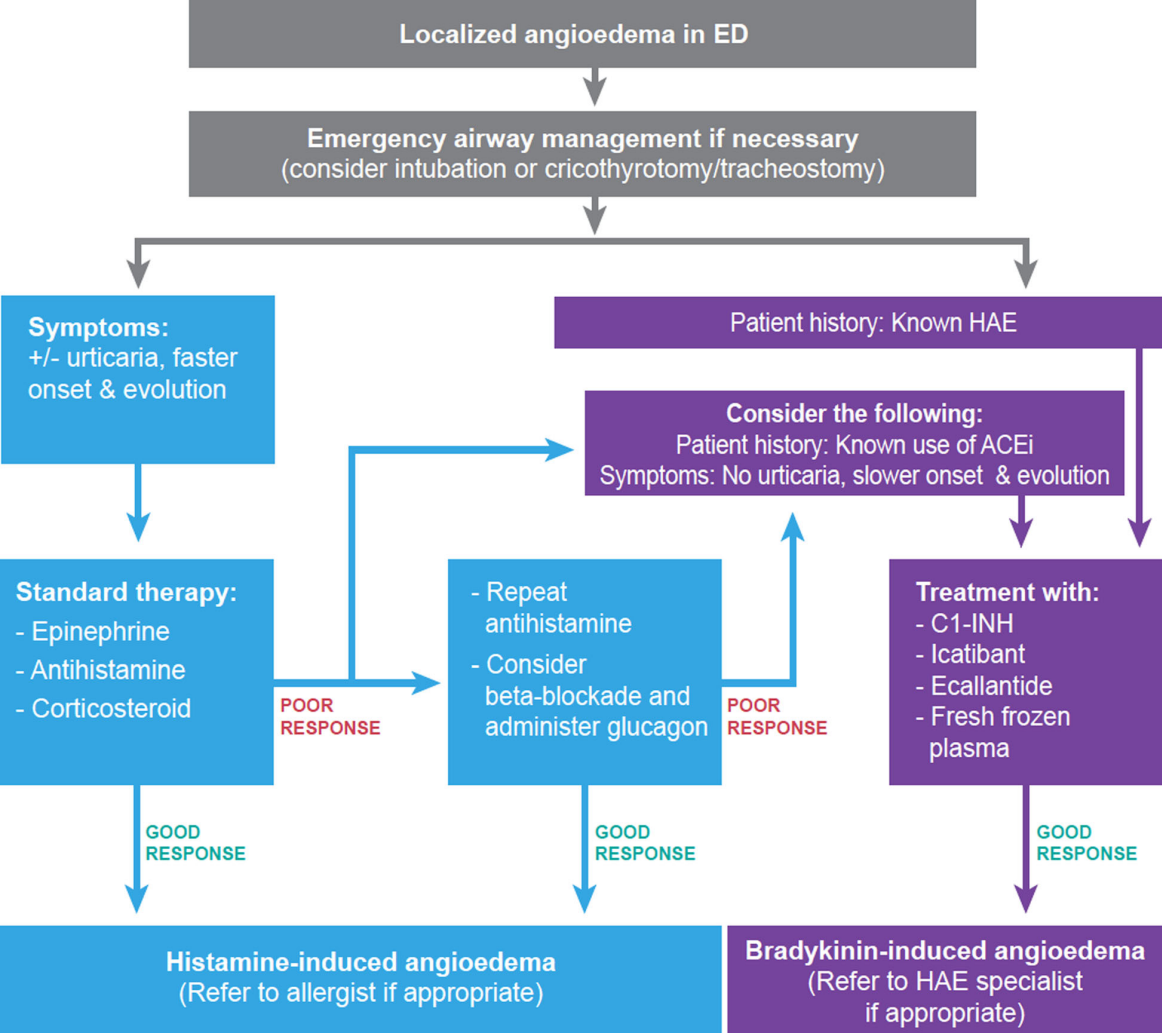
ED response to localised airway angioedema, consistent with published algorithms [1, 3, 4]. When a patient presents at the ED with localised angioedema, airway management should be performed and intubation, tracheotomy or cricothyrotomy procedures should be prepared for worsening symptoms. Unless the patient is known to have HAE or reports prior experience of ACEi- or similar drug-induced angioedema, it is usually difficult to differentiate between histamine- or bradykinin-induced angioedema by clinical presentation alone. Because histamine-induced angioedema is more common, the patient should receive standard treatment (epinephrine, antihistamine and/or corticosteroids). If the symptoms subside, this helps confirm the aetiology as histamine-induced angioedema. Conversely, if symptoms do not resolve or begin to worsen, and beta-adrenergic blockade has been excluded, bradykinin-induced angioedema becomes more likely. Although approved treatments are currently for HAE, some studies have reported that patients with suspected bradykinin-induced angioedema in the ED respond well to these treatments. However, more studies are required to establish their efficacy and use in emergency situations. The patient’s primary care physician should be notified, and the patient should be referred to a specialist if appropriate for long-term management

Consistent with existing guidelines [1, 3–5], the algorithm in Fig. 2 shows the first priority for any patient presenting with angioedema in the ED is assessment of obstruction in the upper airway. All patients with head, neck or lingual angioedema may benefit from flexible fiberoptic nasopharyngolaryngoscopy to determine the extent of swelling. Attempted awake intubation should proceed with an ‘airway double setup’, wherein equipment and bedside expertise are available to initiate emergency tracheotomy or cricothyrotomy if needed [5]. If a pre-existing diagnosis of HAE is known, an appropriate treatment, such as C1-INH may be administered at this point [1–5].

When the underlying cause of angioedema is unknown, standard treatment with epinephrine, antihistamines or corticosteroids should be administered [1, 3, 4]. In the case of a satisfactory response, diagnosis of histamine-induced angioedema is supported, and the patient may be referred to a specialist for long-term management and counselling on the use of an epinephrine autoinjector [1–3, 5]. In the case of non-optimal response, beta-adrenergic blockade should be considered [11]. Otherwise, the index of suspicion rises for a bradykinin-induced aetiology, especially if the patient is on ACE inhibitors (ACEis). Following resolution of a bradykinin-induced episode, patients’ primary care physician should be contacted to discuss medication changes or specialist care, which may be appropriate to confirm the diagnosis supported by laboratory investigation and for long-term management of patients with HAE [1–3].

### How can bradykinin-induced angioedema be treated in the ED?

Table 2 outlines therapeutic options available for HAE in the USA. These treatments are less well studied in acquired- or drug-induced angioedema. Three C1-INH concentrate products are available to inhibit bradykinin biogenesis, as shown in Fig. 1B: plasma-derived C1-INH (Berinert) is approved for treating acute abdominal, facial or laryngeal HAE attacks in adults and paediatric patients; plasma-derived C1-INH (Cinryze) is approved for the routine prophylaxis against angioedema attacks in adults, adolescents and paediatric patients ≥6 years with HAE; and recombinant C1-INH (conestat alfa) is approved for acute attacks in adults and adolescents patients with HAE [12–17]. Icatibant (Firazyr) is a synthetic selective bradykinin receptor antagonist that is approved for acute attacks of HAE in adults, and ecallantide (Kalbitor) is a kallikrein inhibitor that is approved for acute attacks of HAE in patients ≥12 years [18–21]. Although these treatments are approved in the USA, they may not be approved in all other countries. For example, conestat alfa and ecallantide are not licensed in Canada, but may be accessed through Health Canada’s Special Access Programme [22–24]. In situations when these agents are not available, fresh frozen plasma (FFP) containing C1-INH should be considered as an alternative treatment option for HAE attacks [25–27].

**Table 2:** Treatment options for HAE attacks in the USA approved by the Food and Drug Administration (FDA) [12–21, 25–29]

Drug	Indication
Plasma-derived C1-INH (Berinert)	Acute abdominal, facial or laryngeal HAE attacks in adult and paediatric patients
Plasma-derived C1-INH (Cinryze)	Routine prophylaxis against angioedema attacks in adults, adolescents and paediatric patients (6 years of age and older) with HAE
Recombinant C1-INH (Ruconest)	Acute attacks of HAE in adult and adolescent patients with HAE
Icatibant, synthetic bradykinin B2 receptor antagonist (Firazyr)	Acute attacks of HAE in adults 18 years of age and older
Ecallantide, synthetic kallikrein inhibitor (Kalbitor)	Acute attacks of HAE in patients 12 years of age and older
FFP	Deficiency of coagulation factors or plasma protein, when alternative therapies are not available

### How effective are the available treatments for bradykinin-induced angioedema in resolving acute attacks in the ED?

**Fig. 3:**
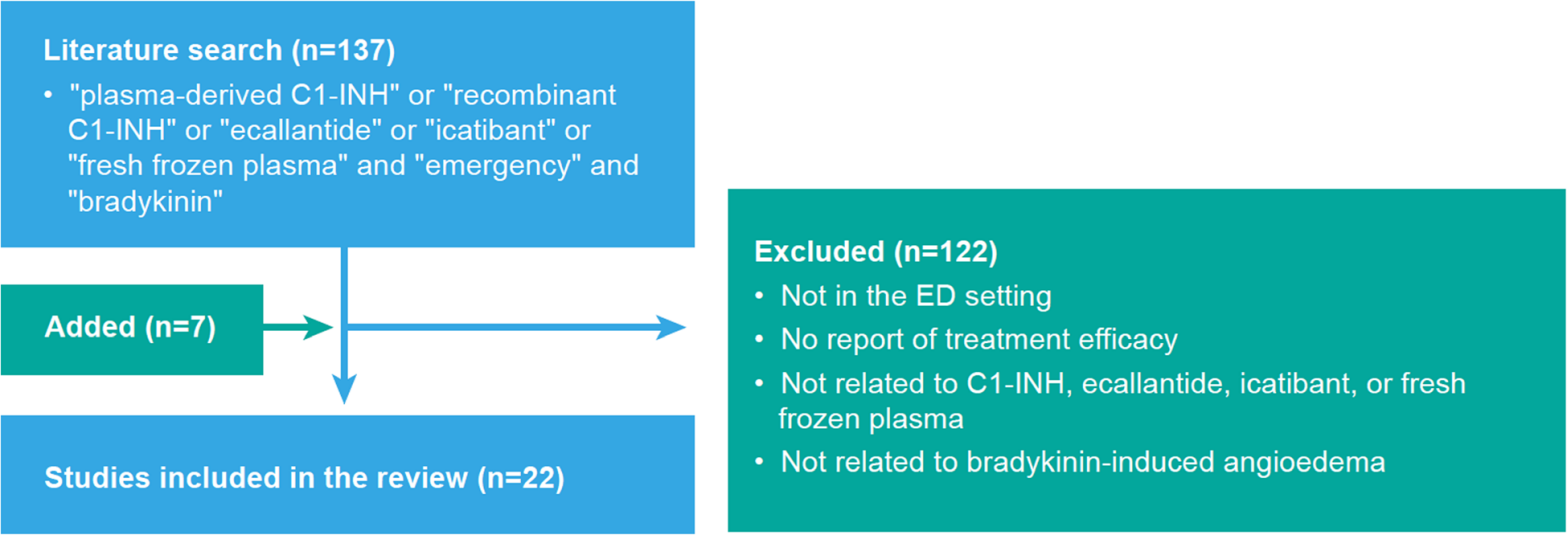
Consort diagram of systematic literature search. Search terms on PubMed generated a total of 137 studies. Those that were not conducted in the ED setting, related to treatment efficacy and/or bradykinin-induced angioedema were excluded (*n* = 122). Seven additional papers relevant to the discussion of the review were included in the analysis. The final 22 studies reported on the effectiveness of current treatments for resolving acute bradykinin-induced angioedema attacks in the ED

Of 22 studies describing the treatment of bradykinin-induced events in the ED (Fig. 3), eight focused on CI-INH, eight on icatibant, one on CI-INH or icatibant, two on ecallantide and three on FFP. These studies include eight prospective studies, one retrospective study, six case series and seven case reports. Most describe drug-induced attacks (16/22); the remainder describe emergency management of HAE attacks. No reports on acquired angioedema were identified. No unexpected safety signals emerged for any of the products studied in these papers.

#### HAE

In a prospective study of 95 patients, 193 emergency laryngeal edema episodes in 42 HAE patients were treated with C1-INH concentrate [30]. The mean episode duration declined 85% with C1-INH (15.3 ± 9.3 h) compared with historical attacks in the same patient population when C1-INH was not used (100.8 ± 26.2 h). Shorter median time to symptom resolution (8.25 h) was also observed in a prospective study with 16 patients treated with C1-INH who experienced 39 emergency laryngeal attacks [31]. Similarly, in two case studies of emergency HAE attacks (one with previously undiagnosed HAE), symptom resolution occurred with C1-INH, whereas standard therapy showed no response [32, 33]. In a retrospective study of 176 emergency episodes amongst 43 patients, 96/98 episodes resolved within 4 h of FFP treatment (IQR: 2–12) [34]. Symptom resolution was also observed amongst three patients treated with FFP in a case series of emergency HAE episodes [35]. These studies demonstrate that C1-INH and FFP are effective in treating HAE attacks in an ED setting.

#### Drug-induced angioedema

Three case reports and one case series describe a total of 14 patients with suspected ACEi-induced angioedema treated in the ED with C1-INH [36–39]. Symptoms resolved in 13 patients. One patient, who experienced a relapse 4 h after treatment, underwent tracheotomy [38]. None of the 10 patients treated with C1-INH from the same case series required intubation or tracheotomy compared to the historical control group who received standard treatment at the same institution (3/47, 6.38% required tracheotomy; 5/47, 10.64% required intubation) [37]. These reports support the idea that C1-INH is effective in treating drug-induced angioedema and that it may avoid the need for invasive interventions.

Studies assessing ED use of icatibant for ACEi-induced angioedema are inconsistent. In two case reports and three case series describing 36 patients with suspected ACEi-induced angioedema, all patients experienced clinical improvement, with symptom regression time of 0.5–7 h following icatibant treatment [40–44]. In a prospective study of 62 patients across EDs of four hospitals, patients were provided with C1-INH, icatibant or standard treatment [45]. C﻿1-INH﻿ or icatibant led to significantly shorter time to symptom relief relative to standard treatment (0.5 h [IQR: 0.5–1.0] C1-INH or icatibant versus 3.9 h [IQR: 2.5–7.0] standard treatment, *p* < 0.0001). In a phase 2 randomised controlled trial (RCT) of 27 patients, complete symptom resolution occurred more quickly with icatibant (8 h [IQR: 3.0–16.0]) compared to the standard therapy (27.1 h [IQR: 20.3–48.0]; *p* = 0.002) [46]. In contrast, a phase 3 RCT of 121 patients and a smaller RCT of 30 patients reported no difference in time to discharge or time to symptom resolution between icatibant and placebo treatment [47, 48]. Meta-analysis indicated that those treated with icatibant experienced a statistically nonsignificant reduction in time to symptom resolution, relative to placebo treatment (mean difference: −7.77 h, 95% CI: −25.18–9.63) [49]. Further investigation is required before establishing the effectiveness of icatibant for drug-induced angioedema in the ED.

Two phase 2 RCTs examined the effectiveness of ecallantide for ACEi-induced angioedema in the ED [50, 51]. The primary endpoint was defined as the proportion of patients discharged within a prespecified time after treatment. In one study, with 4 h as the target time to discharge, 5/24 (21%) patients who received placebo met the endpoint, compared to 8/26 (31%) patients who received ecallantide [50]. In the study with 6 h as the discharge time, 13/18 (72%), 17/20 (85%), 17/19 (89%) and 17/19 (89%) patients receiving placebo, 10, 20 and 60 mg ecallantide, respectively, met the endpoint [51]. None of the differences in discharge time reached statistical significance in these two small studies.

Finally, in a retrospective case series of seven patients with presumed ACEi-induced angioedema refractory to standard treatment, patients were treated with FFP [52]. Symptoms improved in all of these cases, suggesting that FFP may be another effective therapy for drug-induced angioedema.

## Limitations

The studies in this systematic review are small and mostly case series or individual case reports (Table 3). These studies also predominately include drug-induced attacks; not all forms of bradykinin-induced angioedema are represented. Despite these limitations, the literature reviewed here suggests approved HAE therapies may be effective for emergency treatment of various forms of bradykinin-induced angioedema. Awareness of bradykinin-induced angioedema and the therapeutic options for treating it will improve outcomes in the ED.

**Table 3:** Systematic review of the treatment effectiveness for bradykinin-induced angioedema [30–48, 50–52]

Treatment	Author and journal	Study type and population	Description	Findings
**HAE**				
C1-INH	Bork and Barnstedt 2001 [30]	- Prospective study - Series of 95 patients with HAE and functional deficiency of C1-INH	- 42 patients experienced a total of 517 laryngeal episodes over a 20-year observation period - In 193 of these episodes, the patient received C1-INH	- C1-INH was effective in all laryngeal edemas - Duration of laryngeal edema was 15.3 ± 9.3 h in episodes treated with C1-INH versus 100.8 ± 26.2 h in episodes not treated with C1-INH - Mean interval from C1-INH injection to symptom relief was 42.2 ± 19.9 min (ranged: 10 min to 4 h)
	Craig et al. 2010 [31]	- Prospective, multicentre, open-label study - 16 patients with HAE type 1 or II	- 39 emergency laryngeal attacks were treated with C1- INH	- Median time to onset of symptom relief was 15 min—onset of relief observed in at least 95% of attacks within 1 h of treatment - Median time to complete resolution of symptoms was 8.25 h (range: 0.6–48.9 h)
	Gurmen et al. 2017 [32]	- Case report - 34-year-old woman admitted to ED for facial swelling with a known diagnosis of HAE - No continuous use of any medical products	- No response to anaphylaxis treatment - Patient refused FFP - C1-INH was administered	- Symptom regression observed at the end of 10-min C1-INH infusion
	Yigit et al. 2018 [33]	- Case report - 48-year-old-woman admitted to ED with oropharyngeal and facial swelling - No known history of HAE or use of ACEi	- No response to epinephrine and antihistamine - C1-INH was administered	- Swelling resolved completely within 60 min of administration - Diagnosis of HAE type I confirmed after the emergency event
FFP	Pekdemir et al. 2007 [35]	- Case series of three patients presenting at ED with HAE attack - Case 1: 35-year-old woman - Case 2: 21-year-old woman - Case 3: 33-year-old woman	- Case 1: Symptoms persisted after conventional therapy of steroid, antihistamine and epinephrine at two different medical centres before FFP was administered - Case 2: FFP was administered - Case 3: Received FFP and developed allergic reaction so infusion stopped; FFP administration restarted after allergic reaction regressed	- Case 1: Symptoms resolved in first 4 h and patient discharged after 24 h - Case 2: Symptoms resolved within 4 h and patient discharged after 12 h - Case 3: Symptoms began to resolve and patient left ED on her own accord; follow up determined symptoms completely resolved within 5 days
	Wentzel et al. 2019 [34]	- Retrospective study - 43 HAE patients with acute swelling episodes necessitating ED attendance	- 176 acute episodes observed - 98 episodes treated with FFP—observed time to resolution and length of hospital stay	- In 96/98 episodes, FFP led to resolution of symptoms - Median time to resolution was 4 h (IQR: 2–12) - Five transfusion reactions and no deaths occurred (5%)
**Drug-induced angioedema**				
C1-INH	Erickson and Coop 2016 [36]	- Case report - 59-year-old man on > 5 years of ACEi therapy presenting with angioedema of the tongue and difficulty swallowing	- Received conventional therapy of antihistamine, steroids and epinephrine but condition continued to deteriorate - C1-INH was administered	- Patient experienced rapid resolution of symptoms, which avoided airway complications
	Greve et al. 2015 [37]	- Case-control study - Ten adult patients presenting with ACEi-induced angioedema based on patient history and thorough clinical examination - Historical control group included 47 adult patients with ACEi-induced angioedema who had been treated with corticosteroids and antihistamines intravenously at the same institution over the prior 8 years	Assessed time to complete resolution of symptoms following C1-INH	Ten patients in the C1-INH group had shorter time to resolution of symptoms compared with the 47 patients in the control group (10.1 ± 3.0 h versus 33.1 ± 19.4 h; *p* value not reported) - No intubation or tracheotomy was needed in the C1-INH group (0/10), compared to 3/47 patients from the control group requiring tracheotomy, and 2/47 who were intubated
	Leibfried and Kovary 2016 [38]	- Case series - Two cases of ACEi-induced angioedema - Case 1: 60-year-old male with angioedema secondary to lisinopril treatment - Case 2: 64-year-old male with angioedema secondary to enalapril	- Both patients were unsuccessfully treated with conventional treatment (antihistamine, methylprednisolone, epinephrine, FFP)	- Case 1: C1-INH administered with clinical improvement, but symptoms returned 4 h later, and patient underwent tracheotomy - Case 2: Endotracheal tube placement was unsuccessful and C1-INH was administered, resulting in improvement of symptoms
	Rasmussen and Bygum 2013 [39]	- Case report - 63-year-old man taking ACEi presenting with severe angioedema of the tongue and floor of the mouth	- Initially treated with drugs for anaphylaxis (epinephrine, antihistamine and corticosteroids) but angioedema progressed and began to involve soft palate and uvula - C1-INH was administered	- Swelling regressed within 20 min following treatment
Icatibant	Bartal and Stavi 2015 [40]	- Case report - 76-year-old woman presenting with laryngeal edema and dyspnea admitted to ED - Two weeks before the episode, she had begun treatment with enalapril	- Unsuccessfully treated with adrenaline, methylprednisolone, ranitidine and promethazine - Became more dyspneic and agitated, respiratory distress aggravated - During preparation for intubation, icatibant was administered	- Dyspnea was relieved within minutes of treatment, and swelling largely resolved after 30 min - Discharged after 48 h
	Bas et al. 2010 [41]	- Case series with historical control - 8 patients presenting with ACEi-induced angioedema - Retrospectively identified group of 47 patients with similar diagnosis formed the historical comparison group	- Patients were treated with icatibant - Examined for time to first improvement of symptom relief and time to complete symptom relief from treatment - Historical group underwent intensive care interventions and received methylprednisolone and clemastine	- Mean interval to first symptom improvement after icatibant administration was 50.6 ± 21 min - Mean time to complete relief of symptoms in icatibant treatment
				group was 4.4 ± 0.8 h compared with the historical group of 33 ± 19.4 h - In the icatibant treatment group, no patients received tracheal intubation or tracheotomy - In the historical group, 3/47 received tracheotomy and 2/47 were intubated
	Baş et al. 2015 [46]	- Double-blind, multicentre, randomised phase 2 study - 30 patients with ACEi-induced angioedema from two treatment groups were analysed: icatibant (*n* = 15) or placebo with standard treatment of glucocorticoid and antihistamine (*n* = 15) - 27 patients were included in the analysis	- Primary efficacy endpoint was median time to complete resolution of angioedema - Secondary endpoints included time to onset of symptom relief	- Complete resolution of angioedema was 8.0 h (IQR: 3.0–16.0 h) for icatibant and 27.1 h (IQR: 20.3–48.0 h) for placebo (*p* = 0.002)—one patient from the placebo group required tracheotomy—time to onset of symptom relief was 2.0 h (95% CI: 1.0–8.1) for icatibant and 11.7 h (95% CI: 8.0–18.0) for placebo (*p* = 0.03)
	Bova et al. 2015 [42]	- Case series - 13 patients on ACEi seen in ED with angioedema involving face, lips or the upper airways - Initially received standard treatment (antihistamine, corticosteroid, epinephrine) - Lack of response and worsening severity of symptoms	- Patients received icatibant - Time to first and complete symptom relief from treatment were examined and compared with time from previous attacks treated with corticosteroids, antihistamines and/or not treated (10 patients)	- All patients experienced improvement with symptom relief reported at 30 min (IQR: 27.5–70 min) - Complete resolution of symptoms at 5 h (IQR: 4–7 h) - Previous attacks without icatibant treatment had higher median time to complete resolution of 54 h (IQR: 33–63 h; *p* = 0.002) - No patients required tracheal intubation or tracheotomy - All discharged within 24 h
	Crooks et al. 2014 [43]	- Case report - 75-year-old woman presenting with massive tongue and lip swelling secondary to ACEi-induced angioedema	- Awake fibreoptic intubation performed due to impending airway obstruction - No improvement in symptoms after 72 h - Icatibant was administered	- Patient’s trachea successfully extubated 36 h following treatment
	Fok et al. 2015 [44]	- Case series - 13 consecutive ED patients presenting with ACEi-associated upper respiratory tract angioedema - No improvement with adrenaline and/or corticosteroids	- Treated with icatibant - Time from ED presentation to receiving icatibant ranged from 30 min to 3 days (median 3 h)	- Four patients were intubated in the D before or after treatment; three of these were extubated within 24 h of treatment - Eight patients did not require intubation - Time to onset of symptom improvement after icatibant ranged from 15 min to 7 h (median 2 h)
	Sinert et al. 2017 [47]	- Double-blind, multicentre, Phase 3 RCT - 121 patients with ACEi-induced angioedema were randomised to two groups: icatibant (*n* = 61) or placebo (*n* = 60)	- Primary efficacy study endpoint was time to achieving discharge criteria (no difficulty breathing or swallowing, and mild or absent voice change, and tongue swelling)	- No difference in meeting primary endpoint between the two treatment groups - Median time to discharge criteria in the icatibant treatment group was 4.0 h (95% CI: 3.0–5.0) and placebo treatment group was 4.0 h (95% CI: 2.0–5.0) (*p* = 0.63)
	Straka et al. 2017 [48]	- Prospective, double-blind, RCT - 33 patients with ACEi-induced angioedema were randomised to two groups: icatibant (*n* = 15) or placebo (*n* = 18) - 30 patients were included in the analysis	- Primary analyses included time to resolution of symptoms, using survival analysis, and symptom severity (swelling of face, lip, tongue, eyelid) over time using regression analysis	- Survival analysis revealed time to resolution of symptoms was similar between the two treatment groups (*p* = 0.192) - Regression analysis revealed severity of symptoms over time was similar between the two treatment groups (*p* > 0.16)
Icatibant or C1-INH	Javaud et al. 2015 [45]	- Prospective, multicentre, observational study - Consecutive enrollment of 62 patients with ACEi-induced angioedema attacks across ED of four hospitals	- 41 patients were given subcutaneous icatibant (30/41) or C1-INH (11/41), depending upon availability - Reported duration from symptom onset to ED arrival, from symptom onset to treatment decision, from ED arrival to specific treatment, and from specific treatment to onset of symptom relief	- A favourable course was observed in all patients - Median time to onset of symptom relief after C1-INH or icatibant was 0.5 h (IQR: 0.5–1.0), which was significantly shorter than in patients receiving standard treatments (2.9 h (IQR: 2.5–7.0); *p* < 0.0001)
Ecallantide	Bernstein et al. 2015 [50]	- Triple-blinded, phase 2 RCT - Patients experiencing ACEi-induced angioedema not responsive to standard treatment (H1 or H2 antagonists, corticosteroids and epinephrine) within 2 h - 52 patients were randomised to two groups: conventional therapy with ecallantide (*n* = 25) or conventional therapy with placebo (*n* = 27) - 50 patients were included in the analysis	- Primary efficacy study endpoint was achieving discharge criteria from the ED within 4 h after initiating study-related treatment	- Objective discharge criteria met in ≤ 4 h for 5/24 (21%) patients receiving placebo and for 8/26 (31%) of patients receiving ecallantide - Difference in meeting discharge eligibility endpoint criteria between the two groups was not statistically significant
	Lewis et al. 2015 [51]	- Multicentre, double-blind phase 2 RCT - 79 patients with suspected ACEi-induced angioedema in the ED were randomised to placebo with physician- directed conventional therapy or 10, 30 and 60 mg of ecallantide - 76 patients were included in the analysis	- Primary endpoint defined as meeting discharge eligibility criteria within 6 h of study drug administration - Discharge criteria included improvement of angioedema, stable vital signs, absence of stridor, absence of dyspnea or use of accessory muscles during respiration, absence of drooling and ability to drink without difficulty	- The discharge eligibility endpoint was met by 72% of placebo group, and 85%, 89% and 89% of 10, 30 and 60 mg ecallantide groups, respectively - Difference in meeting discharge eligibility endpoint criteria between treatment groups was not statistically significant
FFP	Hassen et al. 2013 [52]	- Case series - Seven patients treated for progressive and refractory presumed ACEi-induced angioedema in the ED	- Refractory to antihistamines, corticosteroids and epinephrine - FFP was administered	- Symptoms improved with treatment in all patients - Avoided intubation in one patient with tongue swelling; stopped progression of facial and lip swelling in five patients; reduced facial and lip swelling in one patient

## Conclusions

Angioedema, when affecting the upper airways, is a challenge for ED physicians because its two primary forms, histamine or bradykinin induced, cannot be readily distinguished on clinical grounds. Histamine-induced angioedema has a faster onset and often presents with urticaria, whilst bradykinin-induced angioedema is slower in onset, with greater incidence of abdominal symptoms. Initial evaluation should focus on airway management and treatment with epinephrine, antihistamine and systemic steroids according to anaphylactic protocols, except for known HAE patients and individuals with a history of drug-induced angioedema. When standard treatment is not effective, assuming proper treatment dosing and beta-adrenergic blockade have been addressed, bradykinin-induced angioedema should be considered and treated accordingly.

Although current approved therapies are indicated for HAE types I and II, Canadian HAE guidelines also recommend their use in patients with normal C1-INH [27]. It is tempting to speculate that the same can be applied to other forms of bradykinin-induced angioedema encountered in the ED. Even though more studies are required to establish this idea, these HAE therapies deserve consideration in emergency situations when standard therapies have failed.

## Data Availability

Not applicable.

## Abbreviations

ACE: Angiotensin-converting enzyme
C1-INH: C1 esterase inhibitor
ED: Emergency department
FDA: Food and Drug Administration
FcεRI: High-affinity IgE receptors
FFP: Fresh frozen plasma
HAE: Hereditary angioedema
HMWK: High-molecular-weight kininogen
IgE: Immunoglobin E
MHC: Major histocompatibility
Th: T-helper
